# Decorin Suppresses Invasion and EMT Phenotype of Glioma by Inducing Autophagy *via* c-Met/Akt/mTOR Axis

**DOI:** 10.3389/fonc.2021.659353

**Published:** 2021-07-27

**Authors:** Yanfei Jia, Qian Feng, Bo Tang, Xiaodong Luo, Qiang Yang, Hu Yang, Qiang Li

**Affiliations:** ^1^Department of Neurosurgery, Second Hospital of Lanzhou University, Lanzhou, China; ^2^Department of Respiratory Medicine, Second Hospital of Lanzhou University, Lanzhou, China

**Keywords:** glioblastoma multiforme, decorin, extracellular matrix, epithelial to mesenchymal transition, autophagy

## Abstract

Decorin exhibits inhibitory effects in tumorigenesis in various types of cancers. The clinical characteristics of 42 patients with GBM were reviewed and analyzed. Lentiviral constructs for decorin overexpression and shRNA-mediated silencing were established for U87MG cells and T98G cells, respectively. The expressions of EMT- and autophagy-associated markers were detected in GBM cell lines. The migration and invasion of the glioma cells were assayed to reflect the malignant behavior of GBM. A mouse xenograft model was used to verify the effect of decorin on autophagy *in vivo*. Reduced expression of decorin in glioma tissues was associated with a poor survival of the patients. Decorin overexpression suppressed cell migration, invasion and attenuated EMT phenotype in glioma cell lines. Further study indicated that decorin inhibited EMT phenotype through the induction of autophagy. The mechanisms include inhibiting the activation of c-Met/Akt/mTOR signaling and regulating the expressions of mesenchymal markers including Slug, vimentin and Twist, and epithelial marker E-cadherin. In addition, decorin overexpression in a mice model can also suppress the GBM invasion and EMT phenotype. In conclusion, decorin suppresses invasion and EMT phenotype of glioma by inducing autophagy *via* c-Met/Akt/mTOR axis.

## Introduction

Glioblastoma multiforme (GBM) is one of the deadliest malignant tumors that occurs in the central nervous system (1). Poor prognosis is commonly found in the GBM patients due to the high invasiveness and resistant to current treatments (2). The epithelial to mesenchymal transition (EMT), a crucial biological process associated with embryonic and post-natal development, has also been reported to regulate tumor aggressive invasion and metastasis in multiple tumors (3, 4), including gliomas (5, 6). In GBM, multiple EMT activators including ZEB1 could induce glioma cells to acquire pseudopodia and higher invasive ability, which are special features of the mesenchymal cells (7). Furthermore, EMT may also initiate the dedifferentiation of the cells, allowing the cells to obtain malignant characteristics including tumor invasive ability and multidrug resistance (8, 9).

Autophagy is an evolutionary conserved homeostatic mechanism *via* degrading misfolded proteins and damaged organelles (10), and dysfunction of autophagy is related to several pathological conditions including cancer occurrence. Autophagy is shown to play double effects on cancer by either inhibiting tumorigenesis *via* protecting the genomic integrity or facilitating tumor growth under metabolic stress and promoting tumor aggressiveness (11). Therefore, the role of autophagy in tumor initiation and progression remains to be further elucidated. Current evidence has indicated that autophagy could maintain cells survival, but an unrestrained autophagy may lead to cell death (12, 13). However, the exact effects of autophagy on the EMT in GBM remain unknown.

Invasion and development of the GBM are promoted by remodeling and degradation of the extracellular matrix (ECM) surrounding the tumor (14). ECM of the central nervous system is composed of a higher content of proteoglycans including tenascin-C and decorin and glycosaminoglycans such as hyaluronic acid (2). These macromolecule components orchestrate the biological behavior of the GBM cells by modulating multiple cellular regulatory signals from the microenvironment of the tumors. Decorin, one of the most intensely studied small leucine-rich proteoglycans (SLRPs), exhibits diverse functions in a variety of pathophysiological processes, such as collagen fibrillogenesis (15, 16), wound healing (17), cell apoptosis, and angiogenesis (18, 19). Decorin is found to exhibit inhibitory effects in tumorigenesis in various types of cancers (20). Current evidence indicates that decorin plays role in tumor cell cycle arrest and cell apoptosis through epidermal growth factor receptor (EGFR) pathway, and decorin also inhibits tumor angiogenesis after formation of a heterodimeric complex with its key receptor Met (21).

In this study, we investigated the role of decorin in autophagy and EMT in GBM, and revealed a molecular mechanism of its inhibitory effects on the malignant behavior of GBM.

## Materials and Methods

### Clinical Specimens and Characteristics

A total of 42 patients with GBM who had received both surgery and chemoradiotherapy were included. The patients were 23 male and 19 female, with a median age of 50.5 years, and ranged 27~69 years. The GBM tissue specimens were collected from the patients had not received other therapies during the surgery in the Second Hospital of Lanzhou University (China). All the fresh specimens were immediately frozen in liquid nitrogen and then stored at -80°C. Tissue sample used for immunohistochemical staining was fixed and embedded in paraffin. The clinical characteristics of the patients were reviewed and analyzed. This study was approved by the Ethics Committee of Second Hospital of Lanzhou University, and the patients signed written informed consent.

### Cell Cultivation and Transfection

The human GBM cell lines including U87MG, T98G, U251, A172 and U118 were acquired from the American Type Culture Collection (ATCC; Rockville, MD). Human normal astrocyte cell line NHAs were acquired from Lonza (Rockland, ME). The cells were cultured in the Dulbecco’s modified Eagle’s medium (DMEM, Gibco, Thermo Fisher Scientific Inc., Waltham, MA) containing 4500 mg/L glucose and 4 mM L-glutamine, and supplemented with 10% fetal bovine serum (FBS, Gibco), 100 units/mL penicillin, and 100 μg/mL streptomycin (Sigma, St. Louis, MO). Cell culture was performed in a humidified atmosphere of 5% CO_2_ and maintained at 37°C.

Lentiviral constructs for decorin overexpression and shRNA-mediated silencing were purchased from GeneChem (Shanghai, China). The vector sequence of decorin shRNA was: 5′-CCGGCCGCATTGCTGATACCAATATCTCGAGATATTGGTATCAGCAATGCGGTTTTTG-3′. The U87MG, T98G and U251 cell lines were cultured in six-well plates at 20~30% cell density one day before transduction. U87MG and U251 cells were transfected with LV-decorin-puromycin at a multiplicity of infection (MOI) of 20, and LV-decorin-shRNA-puromycin was transduced into T98G and U251 cells at a MOI of 40. In addition, non-target virus (LV-puromycin) served as negative control (NC). The DMEM should be replaced by fresh medium 12 h after the incubation. Puromycin was added to the cultured cells to choose the cells transduced with the viruses 48 h after the incubation.

### Reverse Transcription and Quantitative Real-Time Polymerase Chain Reaction (qRT-PCR)

Total RNA was extracted from GBM tissue samples using TRIzol reagent (Gibco, San Diego, CA). 1 μg of RNA was converted to cDNA using reverse transcriptase. qRT-PCR PCR was performed in an ABI PCR instrument (Applied Biosystems, Grand Island, NY) with a Fast SYBR-green Master Mix kit. GAPDH served as an internal control. The relative expression level of PCR product was calculated with the 2^-ΔΔCt^ method. The primers in this assay were decorin, forward: 5′-ATGAAGGCCACTATCATCCTCC-3′ and reverse: 5′-GTCGCGGTCATCAGGAACTT-3′; GAPDH, forward: 5′-GGAGCGAGATCCCTCCAAAAT-3′ and reverse: 5′-GGCTGTTGTCATACTTCTCATGG-3′.

### Primary Glioma Cell Isolation and Cultivation

The primary glioma cells were isolated from three patients (No. 17, No.25 and No.35) and cultured as P017, P025 and P035. The tumor tissues were resected and debrided of the necrotic tissue under sterile conditions, and then digested with 0.25% Trypsin. The cells were harvested after the lysis of red blood cells, washed with phosphate-buffered saline (PBS) and cultured in DMEM supplemented with 10% FBS. The cell medium should be exchanged every two days to remove the non-attached cells until the medium became clarified.

### Western Blot Analysis

The total protein of the cells was extracted using RIPA protein extraction buffer (Beyotime, Shanghai, China) containing protease inhibitor. Proteins were separated using SDS-PAGE gels and transferred to a polyvinylidene fluoride (PVDF) membrane using a iBlot 2 Dry Blotting System (Life technologies, Thermo Fisher Scientific, Waltham, MA). The non-specific reactivity was blocked with nonfat milk at 4°C for one hour. The PVDF membranes were then incubated with primary antibodies including anti-decorin (1:1000), anti-E-cadherin (1:10000), anti-fibronectin (1:1000), anti-vimentin (1:1000), anti-Snail (1:1000), anti-Slug (1:1000), anti-Twist (1:1000), anti-LC3B (1:2000), anti-p62 (1:10000), anti-c-Met (1:1000), anti-p-c-Met (1:1000), anti-Akt (1:1000), anti-p-Akt (1:1000), anti-mTOR (1:10000), anti-p-mTOR, anti-ERK1/2 (1:10000), anti-p-ERK1/2, anti-β-actin (1:5000) and anti-GAPDH (1:5000) at 4°C overnight. These antibodies were from Abcam (Cambridge, MA). The membranes were then washed in TBST and incubated with Horseradish peroxidase-conjugated secondary antibodies (Beyotime, Shanghai, China) for 1 h. A Super ECL Plus Detection reagent (Applygen Technologies, Beijing, China) was used to develop the bands, which were captured by a Tanon-4200 Gel Imaging System (Tanon, Shanghai, China).

### Wound-Healing Assay

The transduced U87MG or T98G cells were seeded in a 12-well plate until confluence. The cell monolayer was manually scratched with a 200 μL-pipette tip to from a straight line without corresponding cells. The wells were gently washed once with PBS to clean the visual field. To decrease the influence of FBS on the cell migration, the FBS concentration in the medium was changed to 0.5%. At least five continuous fields per well were recorded with a Zeiss Axio Observer Z1 inverted microscope (Carl Zeiss, Thornwood, NY) before and 24 h after migration. The ability of cell migration was expressed as the percentage of cell wound closure, which is calculated as (Scratching area - Wound area at 24 h)/Scratching area × 100%. The scratching area and wound area were quantified by the ImageJ software (NIH, Bethesda, MD).

### Cell Invasion Assay

The invasion of transduced U87MG or T98G cells were determined by a BioCoat Matrigel Invasion Chamber (BD Biosciences, Franklin Lakes, NJ). Briefly, cells (1 × 10^5^) in DMEM containing 0.5% FBS were seeded on the upper chamber with Matrigel-coated membrane in a 24-well plate. The bottom chamber was added with DMEM containing 10% FBS as a chemoattractant. After incubating for 24 h, cells on the upper chamber were removed by a cotton swab. The membrane with adhered cells were fixed using 2% paraformaldehyde, and then stained by 0.1% crystal violet PBS solution. Invasive cells were photographed in five random fields per treatment.

### Immunofluorescence Analysis

The GBM cells were fixed with 4% paraformaldehyde in PBS for 15 min, and then permeabilized for 10 min with 0.1% Triton-X100. The cells were blocked with 3% BSA for 30 min. After washed with PBS, the cells were incubated with primary anti-LC3B (1:2000, Abcam) or anti-p62 (1:2000, Abcam) primary antibodies for 1 h at 37°C. The cells were washed and then incubated with corresponding IgG H&L (Alexa Fluor^®^ 488) secondary antibodies (Abcam) for 1 h at room temperature. DAPI was used to stain the cell nucleuses. Images were acquired with an UltraVIEW VoX confocal imaging system (Perkin Elmer, Waltham, MA).

### *In Vivo* Mouse Xenograft Model

Single cell suspension (1 × 10^6^) of U251-decorin-shRNA (shRNA-mediated decorin silencing), U251-decorin (decorin overexpression) or U251-shNC cells were implanted into the subcutaneous tissues in the right abdominal flank of the BALB/c-nu/nu mice. Four weeks after implantation of the cells, the mice were sacrificed. The tumors were fixed in 4% paraformaldehyde, and were then paraffin-embedded for HE staining or immunohistochemical analysis.

### Statistical Analysis

The data in this study were presented as mean ± S.D. Comparisons were performed using two-sided Student’s t-test (two groups), or one-way ANOVA with *post hoc* Tukey’s test (multiple groups). The Kaplan-Meier method was used to plot the survival curves. Survival analysis was performed with the GraphPad Prism 7 Software. P<0.05 is considered significant.

## Results

### Reduced Expression of Decorin in Glioma Tissues Associated With a Poor Survival

**Figure 1:**
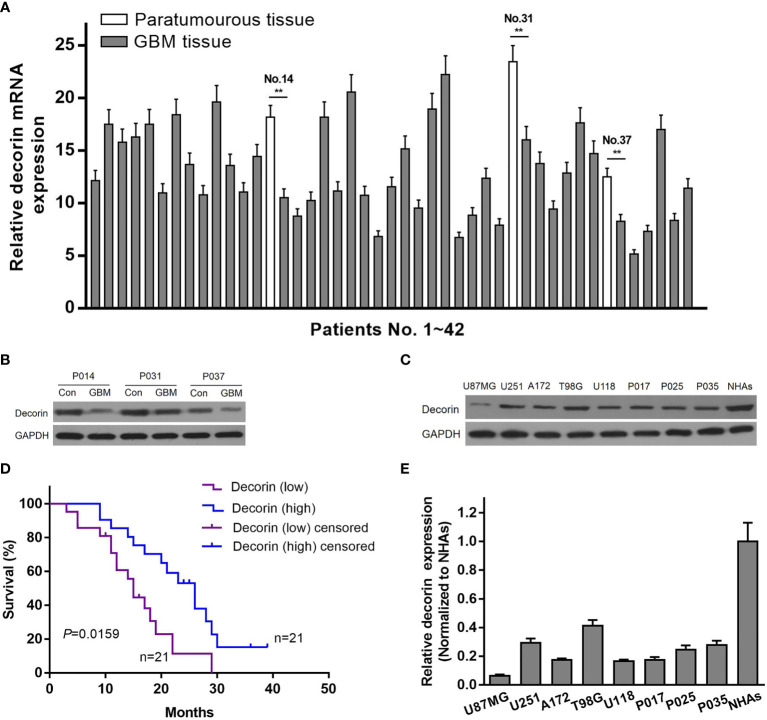
Reduced expression of decorin in glioma tissues is associated with a poor survival. **(A)** The level of decorin expression was detected in 42 GBM samples and 3 paratumorous tissue samples using qRT-PCR. Values are means ± S.D. @ of 3 independent experiments.^**^P <0.01. **(B)** Western blot analysis in three paired tissue samples indicated that decorin was highly expressed in paratumorous tissues, while it had a lower expressions in GBM tissues. **(C)** Kaplan-Meier survival curve according to the levels of decorin expression. The 21 patients with higher decorin expression had significantly better overall survival than those with lower decorin expression (P = 0.0159). **(D)** Western blot analysis showed that decorin was highly expressed in human normal astrocyte cell line NHAs, while it had a lower expressions in established or primary glioma cell lines. **(E)** Measurement data of Western blot results (means ± S.D. @ of 3 independent experiments).

To determine the relationship between decorin and the prognosis of GBM patients, the expression of decorin in GBM tissues was evaluated. qRT-PCR was used to analyze the expression level of decorin in 42 GBM samples and 3 paratumorous tissue samples, and the results were shown in **Figure 1A**. Western blot analysis in three paired tissue samples from some of these patients (No. 14, 31 and 37) indicated that decorin was highly expressed in paratumorous tissues, while it had a lower expressions in GBM tissues (**Figure 1B**). We defined 21 cases with a higher level of decorin expression than the median as the high-expression group or decorin (high). The other 21 cases were included in the low-expression group or decorin (low). According to the Kaplan-Meier survival curve, the 21 patients with higher decorin expression had significantly better overall survival than those with lower decorin expression (P = 0.0159, **Figure 1C**). We further assessed the decorin expression using Western blot on different GBM cells including normal human astrocyte cell line (NHAs), established (U87MG, T98G, U251, A172 and U118) and primary glioma cell lines (P017, P025 and P035). We found a higher decorin expression level in normal astrocyte cell line while at low levels in all glioma cells studied (**Figures 1D, E**).

### Decorin Suppresses Cell Migration, Invasion and Attenuates EMT Phenotype in Glioma Cell Lines

**Figure 2:**
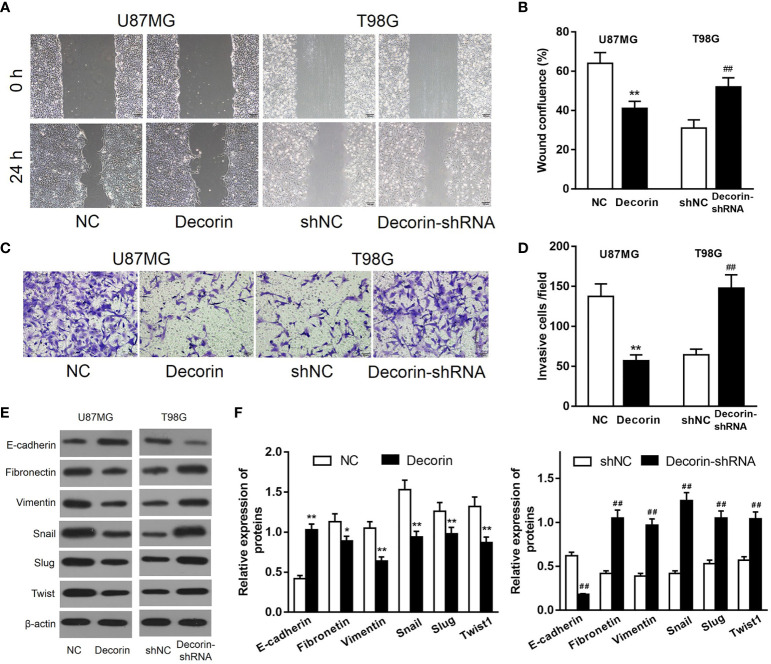
Decorin inhibits cell migration, invasion and ameliorates EMT phenotype in glioma cell lines. **(A)** Wound-healing assay of U87MG and T98G cells with decorin overexpression and decorin silencing. Decorin overexpression dramatically decreased the cell migration in U87MG-decorin cells compared with that in U87MG-NC cells. T98G-decorin-shRNA cells showed a significantly increased cell migration compared to the T98G-shNC cells. **(B)** Measurement data of cell migration results (means ± S.D. @ of 3 independent experiments). **(C)** Overexpression of decorin significantly decreased the number of invasive U87MG cells. However, silencing of decorin significantly promoted cell invasion in T98G cells. **(D)** Measurement data of cell invasion results (means ± S.D. @ of 3 independent experiments). **(E)** Evaluation of the effects of decorin on the EMT phenotype. Western blot results showed that overexpression of decorin significantly increased the expression of E-cadherin, but suppressed the mesenchymal markers vimentin and fibronectin, and inhibited the expressions of EMT-related proteins Snail, Slug and Twist. In contrast, decorin-silencing down-regulated the expression of E-cadherin, and up-regulated the mesenchymal markers and EMT-related proteins. **(F)** Measurement data of Western blot results (means ± S.D. @ of 3 independent experiments). ^*^P < 0.05, ^**^P < 0.01 *vs.* NC; ^##^P < 0.01 *vs.* shNC.

Because a low expression of decorin was correlated with a poor prognosis in patients with GBM, we then investigated whether decorin played a functional role in glioma cells. Lentiviruses-mediated overexpression of decorin in U87MG cells (U87MG-decorin), or expression of short hairpin RNAs (shRNA) to knock down decorin in T98G cell lines (T98G-decorin-shRNA) were performed. Wound-healing assay revealed that decorin overexpression dramatically decreased the cell migration in U87MG-decorin cells compared with that in U87MG-NC cells. In addition, T98G-decorin-shRNA cells showed a significantly increased cell migration compared to the T98G-shNC cells (**Figures 2A, B**). Consistently, overexpression of decorin significantly decreased the number of invasive U87MG cells. However, silencing of decorin significantly promoted cell invasion in T98G cells (**Figures 2C, D**).

To evaluate the potential effects of decorin on the regulation of EMT phenotype, the expression of EMT-associated markers was detected. The result of Western blot indicated that overexpression of decorin significantly increased the expression of E-cadherin, which was expressed in the neural tissue, but suppressed the mesenchymal markers vimentin and fibronectin, and the expressions of EMT-related proteins Snail, Slug and Twist were inhibited as well. In contrast, decorin-shRNA down-regulated the expression of E-cadherin, and up-regulated the mesenchymal markers and EMT-related proteins (**Figures 2E, F**). These results suggested that decorin significantly inhibited the occurrence of EMT, which could be promoted by decorin-shRNA.

### Decorin Induces Autophagy in Glioma Cell Lines

**Figure 3:**
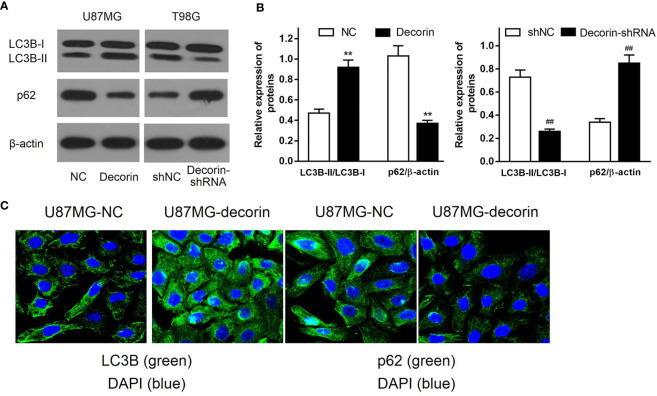
Decorin stimulates autophagy in glioma cell lines. **(A)** Western blot analysis of autophagy related protein indicated that increased LC3B-I to LC3B-II conversion and reduced expression of p62 were both found in U87MG-decorin cells. Decorin-silencing decreased the ratio of LC3B-II/LC3B-I and elevated the expression of p62 in T98G cells. **(B)** Measurement data of Western blot results (means ± S.D. @ of 3 independent experiments). ^**^P < 0.01 *vs.* NC; ^##^P < 0.01 *vs*. shNC. **(C)** Immunofluorescence analysis of LC3B and p62 in U87MG cells, in response to overexpression of decorin.

To determine the potential mechanism of the inhibitory effects of decorin overexpression on cell invasion and EMT, the levels of autophagy-related proteins were detected. Increased LC3B-I to LC3B-II conversion and reduced expression level of autophagy cargo protein p62 were both found in U87MG-decorin cells, indicating that autophagy was activated by decorin overexpression. In addition, decorin-silencing decreased the ratio of LC3B-II/LC3B-I and elevated the expression of p62 in T98G cells compared with those in shNC cells (**Figures 3A, B**). We then performed immunofluorescence assay to further detect the distribution of LC3B and p62 in U87MG cells. In consistent with western blot analysis, the results showed increased numbers of LC3B protein spots and decreased expression of p62 in the cytoplasm of decorin-overexpressed cells, suggesting that decorin overexpression could promote the cell autophagy (**Figure 3C**).

### Decorin Inhibits EMT Phenotype Through the Induction of Autophagy in Glioma Cell Lines

**Figure 4:**
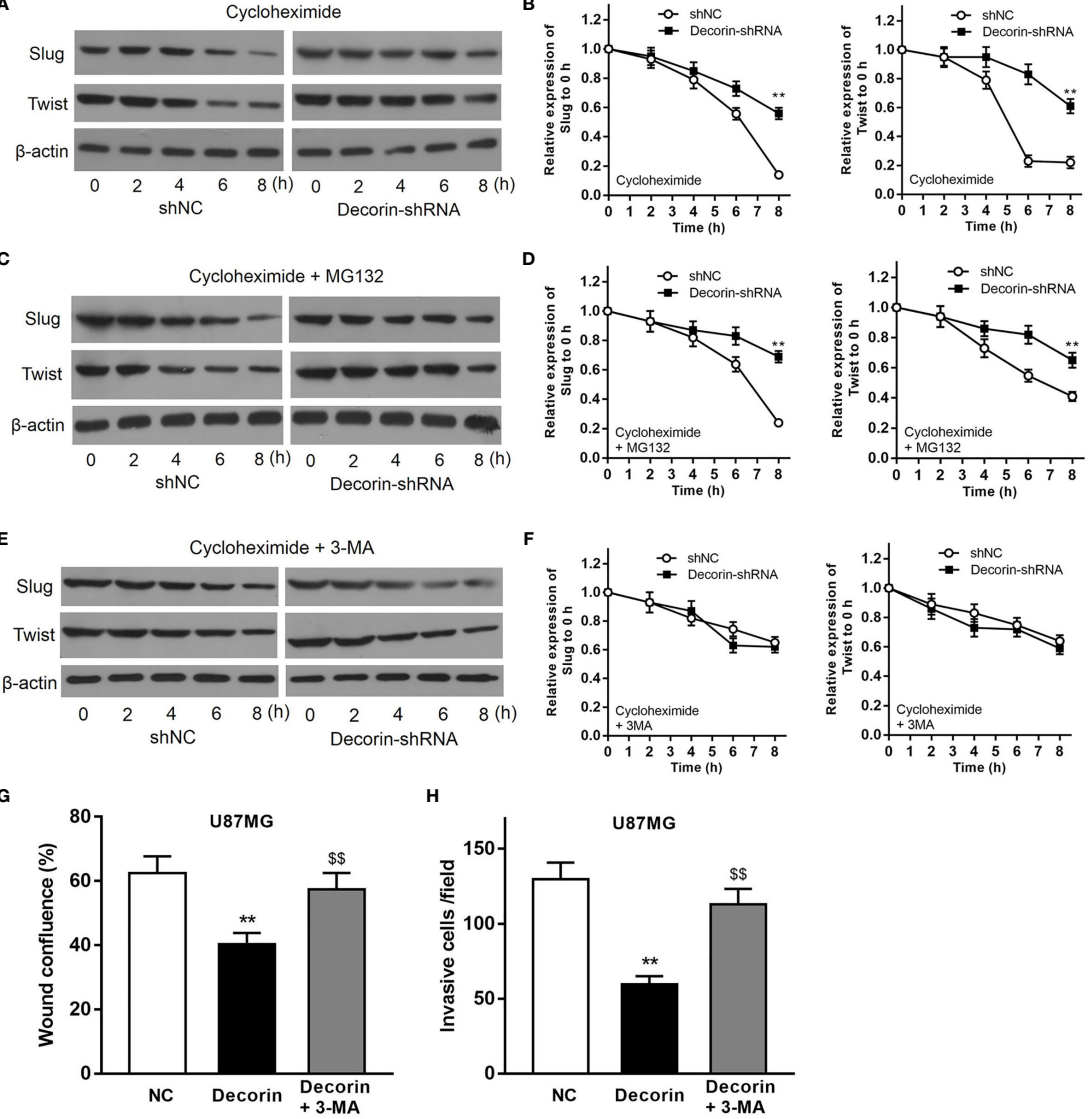
Decorin inhibits EMT phenotype through the induction of autophagy in glioma cell lines. **(A)** T98G cells were infected with LV-decorin-shRNA vector. Cells were treated with 100 μg/mL of cycloheximide. Western blot results showed that the degradation of Slug and Twist in T98G-shNC cells were much rapid than that in T98G-decorin-shRNA cells. **(B)** Measurement data of **(A)** (means ± S.D. @ of 3 independent experiments). ^**^P<0.01 *vs*. shNC. **(C)** Treatment of the cells with both cycloheximide and 10 μM of MG132 could not change these effects between groups. **(D)** Measurement data of **(C)** (means ± S.D. @ of 3 independent experiments). ^**^P < 0.01 *vs.* shNC. **(E)** The degradation rates of Slug and Twist were similar between T98G-shNC and T98G-decorin-shRNA cells after the cells were treated with cycloheximide and 10 mmol/L 3-MA. **(F)** Measurement data of **(E)** (means ± S.D. @ of 3 independent experiments). **(G, H)** 3-MA treatment could block the effects of decorin overexpression-induced inhibition of migration and invasion of U87MG cells. (means ± S.D. @ of 3 independent experiments).). ^**^P < 0.01 *vs.* NC; ^$$^P < 0.01 *vs.* Decorin.

To further discover the mechanisms underlying the inhibitory effects of decorin on the cell invasion and EMT phenotype, we examined whether autophagy–lysosome degradation system contributed to decorin-induced down-regulation of Slug and Twist expression, both of which play critical roles in the regulation of EMT. T98G-shNC or T98G-decorin-shRNA cells were treated with cycloheximide, a widely used protein synthesis inhibitor, or cycloheximide combined with proteasome inhibitor MG132, to block *de novo* synthesis and the ubiquitin-proteasome degradation of Slug and Twist. The result indicated that the degradation of Slug and Twist in T98G-shNC cells were much rapid than that in T98G-decorin-shRNA cells (**Figures 4A, B**). However, treatment of cells with both cycloheximide and MG132 could not change these effects between groups (**Figures 4C, D**). These data indicate that the decorin-mediated degradation of Slug/Twist does not depend on the ubiquitin-proteasome system.

The cells were then treated with cycloheximide and 3-MA, an inhibitor of autophagy. The result of Western blot indicated that the degradation rates of Slug and Twist were similar between T98G-shNC and T98G-decorin-shRNA cells, suggesting that 3-MA significantly attenuate the degradations of these proteins (**Figures 4E, F**). Therefore, a decorin-dependent mechanism is involved in degradation of Slug/Twist *via* activating the autophagy-lysosome system. Furthermore, 3-MA treatment could block the effects of decorin overexpression-induced inhibition of migration and invasion of U87MG cells (**Figures 4G, H**). Collectively, these findings suggest that decorin inhibits EMT phenotype through the induction of autophagy in glioma cell lines.

### Decorin Suppresses EMT *via* c-Met/Akt Axis in Glioma Cells

**Figure 5:**
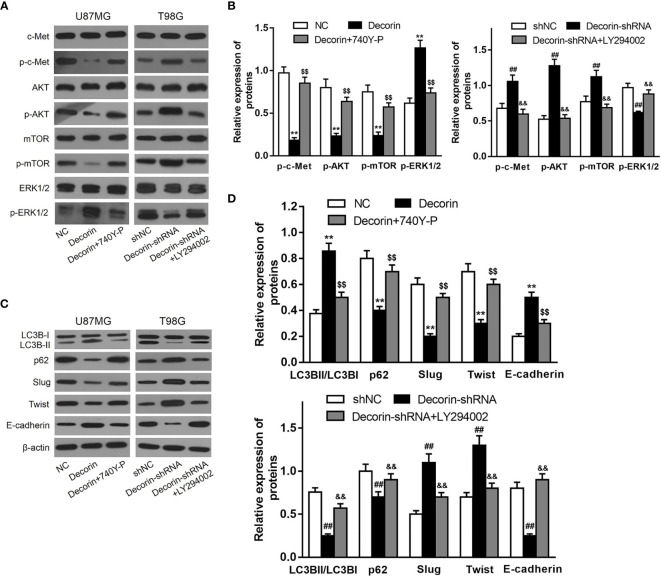
Decorin suppresses EMT *via* c-MET/Akt/mTOR signaling pathway in glioma cells. **(A)** U87MG and T98G cells were infected with decorin-overexpressing lentivirus and decorin-shRNA lentivirus, respectively. U87MG were treated with 740Y-P, a PI3K activator, and T98G cells with the inhibitor LY294002. The cells were then harvested and lysed for the detection of p-c-Met, c-Met, p-Akt, Akt, p-mTOR, mTOR, p-ERK1/2 and ERK1/2 by western blot. The phosphorylated levels of c-Met, Akt and mTOR were notably down-regulated and the level of p-ERK1/2 was increased in decorin-overexpressed U87MG cell lines compared with the U87MG-NC cells. In contrast, the phosphorylated levels of c-Met, Akt and mTOR were significantly up-regulated, whereas the level of p-ERK1/2 was decreased in T98G-decorin-shRNA cells. The U87MG-decorin cells, the expression levels of p-c-Met, p-Akt and p-mTOR were significantly increased, and p-ERK1/2 level was decreased after treatment with 740Y-P, but these expression changes were reversed in the T98G-decorin-shRNA cells after treatment with LY294002. **(B)** Measurement data of **(A)** (means ± S.D. @ of 3 independent experiments). ^**^P < 0.01 *vs.* NC; ^$$^P<0.01 *vs*. Decorin; ^##^P < 0.01 *vs.* shNC; ^&&^P < 0.01 *vs.* Decorin-shRNA. **(C)** LC3B-II/LC3B-I expression ratio and E-cadherin was decreased in the decorin-overexpressed U87MG cells after 740Y-P treatment, but the levels of P62, Slug and Twist were augmented in these cells. Contrarily, LY294002 treatment led to reduced expressions of p62, Slug and Twist, and up-regulated the expressions of p-ERK1/2, E-cadherin and LC3B-II/LC3B-I conversion. **(D)** Measurement data of **(C)** (means ± S.D. @ of 3 independent experiments). ^**^P < 0.01 *vs.* NC; ^$$^P < 0.01 *vs.* Decorin; ^##^P < 0.01 *vs.* shNC; ^&&^P < 0.01 *vs.* Decorin-shRNA.

As we had revealed decorin-mediated degradation of Slug/Twist, we then examined whether decorin directly inactivate EMT-prompting signal pathways. Mounting evidence has shown that decorin exert its oncosuppressive function as an endogenous pan-receptor tyrosine kinase inhibitor. Meanwhile, pathways mediated by tyrosine kinase receptors have been reported to participate in the activation of EMT-like related genes to promote GBM dissemination. Among these RTKs, the hepatocyte growth factor (HGF) binding receptor tyrosine kinase receptor c-Met is highly activated during GBM progression. The activated receptor is associated with a disassembly of adherent junction, resulting in increased cell migration and promoting EMT (22). Current evidence indicates that autophagy can be negatively regulated by PI3K/Akt protein pathway and positively regulated by ERK1/2 protein pathway (23). Thus, we sought to examine whether c-Met/PI3K/Akt axis is involved in decorin-induced EMT inhibition in glioma cells. First, we evaluated the phosphorylated levels of c-Met, Akt and mTOR in T98G-decorin-shRNA and U87MG-decorin cells, and the results indicated that phosphorylated levels of c-Met, Akt and mTOR were notably down-regulated by Western blot analysis. The level of p-ERK1/2 was increased in decorin-overexpressed U87MG cell lines compared with the U87MG-NC cells (**Figures 5A, B**). In contrast, the phosphorylated levels of c-Met, Akt and mTOR were significantly up-regulated, whereas the level of p-ERK1/2 was decreased in T98G-decorin-shRNA cells (**Figures 5A, B**). Although the carcinostatic function of decorin is specific, further investigation is urgently needed to illustrate the mechanism through which decorin affects the EMT and autophagy in tumors *via* the c-Met/Akt/mTOR and ERK1/2 signaling pathway. Thus, we used LY294002, a PI3K inhibitor, and the activator 740Y-P to treat the cells showing stable decorin knockdown or overexpression and evaluated the phosphorylated levels of c-Met, Akt, mTOR and ERK1/2, and the expressions of autophagy- and EMT-related markers by Western blot analysis. The results showed that in the U87MG-decorin cells, the expression levels of p-c-Met, p-Akt and p-mTOR were significantly increased, and p-ERK1/2 level was decreased after treatment with 740Y-P, but these expression changes were reversed in the T98G-decorin-shRNA cells after treatment with LY294002 (**Figures 5A, B**). In addition, the LC3B-II/LC3B-I expression ratio and E-cadherin was decreased in the decorin-overexpressed U87MG cells after 740Y-P treatment, but the levels of P62, Slug and Twist were augmented in these cells (**Figures 5C, D**). Contrarily, LY294002 treatment led to reduced expressions of p62, Slug and Twist, and up-regulated the expressions of p-ERK1/2, E-cadherin and LC3B-II/LC3B-I conversion, resulting in activation of ERK1/2 signaling and thereby the induction of autophagy and EMT inhibition (**Figures 5A, C, D**). Therefore, the regulatory effects of decorin on autophagy and the EMT can be partially attributed to the inhibition of PI3K-Akt-mTOR and activation of ERK1/2 signaling pathways.

### Decorin Inhibits EMT Phenotype in Glioma Cells *via* Activation of Autophagy *In Vivo*

**Figure 6:**
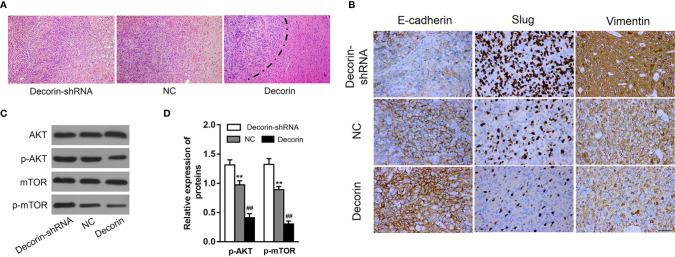
Decorin overexpression suppresses GBM invasion and EMT phenotype. **(A)** The result of HE staining showed that U251-shNC tumor or U251-decorin-shRNA tumor tended to invade the neighboring normal tissue, however, the U251-decorin tumor kept a relative smooth edge. **(B)** The expression of mesenchymal markers including Slug and vimentin were up-regulated greatly in U251-decorin-shRNA tumors. The expressions of these proteins were decreased in U251-shNC tumors, and further significantly decreased in U251-decorin tumors. Conversely, the epithelial marker E-cadherin was down-regulated in U251-decorin-shRNA tumors, and the expression was increased in U251-shNC tumors, and further significantly increased in U251-decorin tumors. **(C)** The phosphorylated levels of Akt and mTOR were notably up-regulated in U251-decorin-shRNA tumors compared to those in U251-shNC tumors, and these phosphorylated levels were further decreased in U251-decorin tumors. **(D)** Measurement data of **(C)** (means ± S.D. @ of 3 independent experiments). ^**^P < 0.01 *vs.* Decorin-shRNA; ^##^P < 0.01 *vs.* NC.

Along with the *in vitro* cellular data, we tested whether decorin could reduce tumor invasion and inhibit autophagy and the EMT phenotype *in vivo*. The role of decorin in a nude mouse model was analyzed. Lentiviral constructs for shRNA-mediated decorin silencing (U251-decorin-shRNA) and decorin overexpression (U251-decorin) were established in U251 cells. Compared to the U251-decorin tumor, U251-shNC and U251-decorin-shRNA tumors exhibited clear characteristics of invasion. The result of HE staining showed that U251-shNC tumor or U251-decorin-shRNA tumor tended to invade the neighboring normal tissue, however, the U251-decorin tumor kept a relative smooth edge (**Figure 6A**). The expression of mesenchymal markers including Slug and vimentin were up-regulated greatly in U251-decorin-shRNA tumors. The expressions of these proteins were decreased in U251-shNC tumors, and further significantly decreased in U251-decorin tumors. Conversely, the epithelial marker E-cadherin was down-regulated in U251-decorin-shRNA tumors, and the expression was increased in U251-shNC tumors, and further significantly increased in U251-decorin tumors. These data suggested that EMT was induced by decorin inhibition *in vivo* (**Figure 6B**). In addition, the phosphorylated levels of Akt and mTOR were notably up-regulated in U251-decorin-shRNA tumors compared to those in U251-shNC tumors, and these phosphorylated levels were further decreased in U251-decorin tumors (both P<0.01, **Figures 6C, D**). These data suggested that Akt/mTOR pathway was inhibited by decorin in the implantated tumors, which was consistent with the invasion data *in vivo*.

## Discussion

Proteoglycans are a macromolecule family with complex structures and high heterogeneity. Proteoglycans have a protein core and at least one covalently attached glycosaminoglycan chain. This unique structure provides the proteoglycans with the ability to regulate multiple pathophysiological processes including tumorigenesis (24). Decorin, which belongs to the small leucine-rich proteoglycan (SLRP) family, is a key component of ECM structure and function. Recent studies revealed that decorin exhibits potent oncosuppressive activities in multiple tumors (20, 21) through extracellular and intracellular mechanisms. In this study, we investigated the role of decorin in autophagy and EMT in GBM, and revealed that the high expression level of decorin correlates with the better overall survival of GBM patients. This differential expression was also observed in cultured cells that a higher decorin expression found in normal astrocyte cell line NHAs while it was at low levels in established glioma cells (U87MG, T98G, U251, A172 and U118) and the glioma cells isolated from three GBM patients. Notably, although GAPDH is a normally used reference gene for RT-qPCR assay, Rydbirk et al. reported that more stable reference gene such as UBE2D2 or RPL13 may be better for assessing gene expression in nerve tissues (25). In addition, we found that decorin regulates autophagy and the EMT phenotype through c-Met/Akt/mTOR and ERK1/2 pathway.

In addition to playing an essential role in embryonic development, EMT has also been implicated to modulate tumor invasion and metastasis in numerous tumors (26, 27) including GBM (28). Recent studies have shown that decorin not only exerts its actions within the tumor stroma, but also acts as a multifunctional signaling molecule in numerous pathological conditions such as hepatic fibrosis (29), immunomodulation (30, 31), obesity (32) and tumor initiation and progression (20, 33, 34). In this study, lentiviral constructs for decorin overexpression and shRNA-mediated silencing were established for U87MG cells and T98G cells, respectively. We observed that decorin overexpression in U87MG cells suppressed cell migration and invasion, accompanied by the reversion of EMT phenotype, which is characterized by down-regulation of mesenchymal marker fibronectin, vimentin, Snail, Slug and Twist, and up-regulation of epithelial marker E-cadherin. In contrast, silencing of decorin in T98G cells promoted cell migration, invasion and EMT process.

The current study further found that inhibition of the EMT by decorin was mediated by the activation of autophagy. Multiple autophagy-associated proteins are involved in this complicated process. In the present study, it was shown that overexpression of decorin in U87MG cells up-regulated the conversion of LC3B-II, and reduced the expression level of autophagy cargo protein p62. Immunofluorescence assay also confirmed the increased level of LC3B and decreased p62 level in U87MG cells transfected with decorin. These results suggest that decorin induces autophagy in human glioma cells. In addition, in U87MG cells normally lacking decorin expression, ectopic overexpression of decorin led to the autophagy-lysosome dependent degradation of Slug and Twist, two main promotors of EMT, to attenuate the EMT process in U87MG cells. In contrast, in T98G cells normally expressing decorin, silencing of decorin induced the accumulation of Slug and Twist, and activated the EMT process. Furthermore, autophagy inhibitor (3-MA) treatment could block the effects of decorin overexpression-induced inhibition of migration and invasion of U87MG cells. Thus, our study suggests that decorin shows its GBM-inhibitory effects through induction of autophagy activity to suppress the EMT process.

Recent reports have revealed that decorin can act as an anti-metastatic effector, suppressing migration and invasion of cancer cells (35–37). In a mice model of colon carcinoma, decorin inhibits the growth and migration of cancer cells through regulating the level of E-cadherin (38). The inhibitory effects of decorin on oncogenesis are associated with the activation of receptor complex (34). Using a discovery tool, such as a phosphotyrosine RTK array, a RTK, Met or HGF receptor was found to be specifically activated by soluble decorin proteoglycan or decorin protein core (39). Previous studies indicated that Met is a crucial receptor of decorin, and relays multiple oncosuppressive signals (39, 40). However, the relation between decorin, EMT and c-Met/Akt/mTOR axis remains unclear in glioma cells. Here, we characterized a specific signaling pathway in human glioma cells. Decorin overexpression in U87MG cells blocked the phosphorylation of c-Met, Akt and mTOR followed by downregulation of Slug, Twist and p62, and an increase of E-cadherin, LC3B-II to LC3B-I conversion. These effects were reversed when treated with PI3K activator 740Y-P. In contrast, the phosphorylation of c-Met, Akt and mTOR were inhibited, followed by downregulation of Slug, Twist and p62, and an increase of E-cadherin, LC3B-II to LC3B-I conversion in the decorin-knockdown T98G cells after treatment with the PI3K inhibitor LY294002.

Although implantation of the tumor cells into the brain is closer to the growth environment in the human, complicated operation and higher incidences of infection and death are major drawbacks of this model. Furthermore, it is also difficult to consecutively observe the growth of tumor. Thus, in this study, we used a mice model of subcutaneous implantation of tumor cells due to easy operation and observation. The *in vivo* result indicated that decorin overexpression can suppress the GBM invasion and EMT phenotype. In addition, the phosphorylated levels of Akt and mTOR were significantly decreased in U251-decorin tumors compared to those in both U251-decorin-shRNA tumors and U251-shNC tumors. Therefore, we demonstrated that decorin inhibits migration, invasion and EMT by the suppressing the c-Met/Akt/mTOR signaling pathway.

In conclusion, this study provides evidence that overexpressed decorin attenuated the EMT, migration and invasion of human glioma cells. The mechanisms include inhibiting the activation of c-Met/Akt/mTOR signaling and regulating the expression of the important mesenchymal markers including Slug, vimentin and Twist, and epithelial marker E-cadherin. These findings provide a basis for the action of decorin regulation in the GBM.

## Data Availability Statement

The raw data supporting the conclusions of this article will be made available by the authors, without undue reservation.

## Ethics Statement

The animal study was reviewed and approved by Second Hospital of Lanzhou University.

## Author Contributions

QL conceived and designed the current study and contributed to writing the manuscript. YJ and QF performed the experiments. BT, XL, QY, and HY analyzed and interpreted the data. All authors read and approved the final manuscript.

## Conflict of Interest

The authors declare that the research was conducted in the absence of any commercial or financial relationships that could be construed as a potential conflict of interest.

## Publisher’s Note

All claims expressed in this article are solely those of the authors and do not necessarily represent those of their affiliated organizations, or those of the publisher, the editors and the reviewers. Any product that may be evaluated in this article, or claim that may be made by its manufacturer, is not guaranteed or endorsed by the publisher.
